# Methylation-associated downregulation of CRABP1 marks an aggressive malignant epithelial cell state in thyroid carcinoma

**DOI:** 10.3389/fcell.2026.1974822

**Published:** 2026-10-14

**Authors:** Tiancheng Fu, Yudong Li, Guoxiang Huang, Xiarong Hu, Guangfeng Luo, Chengcheng Wang, Yanze Wang, Ziyang Ding, Jinghua Zhou, Zhen Li

**Affiliations:** 1 Department of Thyroid Surgery, The Tenth Affiliated Hospital, Southern Medical University (Dongguan People’s Hospital), Dongguan, Guangdong, China; 2 Northern Jiangsu People’s Hospital Affiliated to Yangzhou University, Yangzhou, Jiangsu, China; 3 The Fourth General Surgery Department of the Fourth Affiliated Hospital of China Medical University, Shenyang, Liaoning, China; 4 Liaoning Fangda General Hospital, Shenyang, Liaoning, China

**Keywords:** CRABP1, decitabine, DNA methylation, epithelial-mesenchymal transition, papillary thyroid carcinoma, single-cell RNA sequencing, thyroid carcinoma

## Abstract

Papillary thyroid carcinoma (PTC) is the most common thyroid malignancy, yet molecular features that distinguish indolent tumors from those with progression-associated clinicopathological features remain limited. Here, we integrated single-cell RNA sequencing of 17 PTC samples with copy-number inference and identified a CRABP1-positive malignant epithelial cell population that retained epithelial features (CDH1, KRT19) and exhibited a distinct transcriptional program. In TCGA-THCA bulk data, lower CRABP1 expression was associated with lymph-node metastasis and pathological T-category variation. Functional experiments in complementary thyroid carcinoma cell-line models showed that CRABP1 knockdown increased migration and invasion and was accompanied by an EMT-related transcriptional shift, whereas CRABP1 overexpression showed the opposite pattern. Public methylation analyses showed higher aggregate promoter-region methylation in tumors, whereas methylation of cg02276974, an intron-2/gene-body CpG, was weakly inversely associated with CRABP1 expression (r = −0.2069, R^2^ = 0.0428; P < 0.001). Decitabine altered migration, invasion, Vimentin-associated, and xenograft phenotypes in CRABP1-manipulated models. Collectively, these results identify a CRABP1-positive malignant epithelial cell state associated with clinicopathological features in PTC and support an invasion-associated suppressive role for CRABP1 in thyroid carcinoma models. The methylation and decitabine findings define a methylation-associated pharmacological context; they do not establish CRABP1-specific or locus-specific epigenetic causality.

## Introduction

Papillary thyroid carcinoma (PTC) is the most common histological subtype of thyroid malignancy and is generally associated with favorable long-term survival. However, the clinical challenge in contemporary PTC management is to distinguish indolent lesions from tumors with genuine progression potential. Recent population-level evidence indicates that many newly diagnosed PTCs may reflect overdiagnosis, whereas active surveillance studies show that a subset of low-risk PTCs still exhibits measurable growth or newly detected lymph node metastasis during follow-up (Yoon et al., 2024; Francis et al., 2026). In addition, occult lateral lymph node metastasis remains clinically relevant even among patients initially classified as clinically node-negative, underscoring the need for molecular features that refine risk stratification beyond conventional clinicopathological assessment (Fei et al., 2024).

PTC is characterized by substantial tumor heterogeneity. Malignant epithelial-cell states can differ in differentiation status, invasive potential, and interactions with stromal and immune components of the tumor microenvironment. Recent analyses of PTC ecosystems have identified malignant thyrocyte phenotypes associated with follicular differentiation, partial mesenchymal-like programs, dedifferentiation, lymph node involvement, and microenvironmental remodeling (Pu et al., 2021; Yan et al., 2021). Multi-omics studies have further linked malignant-cell differentiation states with prognosis-related molecular programs, supporting the view that tumor-cell state is an important layer of PTC biological diversity (Ji et al., 2025). Defining such malignant epithelial-cell states may therefore provide a more precise context for evaluating candidate regulators of PTC progression.

Cellular retinoic acid binding protein 1 (CRABP1), a retinoid-signaling-associated molecule, has recently attracted attention as a thyroid-cancer-relevant regulator. CRABP1 expression was reported to be reduced in thyroid cancer, and experimental restoration of CRABP1 suppressed migratory and invasive activity while attenuating mesenchymal phenotypic features in thyroid cancer models (Hsu et al., 2023). In RAS-mutant thyroid tumors, transcriptomic analysis also identified CRABP1 among a gene set associated with invasive behavior, further suggesting a relationship between reduced CRABP1 expression and tumor invasiveness (Condello et al., 2025). These findings support the rationale for examining CRABP1 in relation to malignant epithelial-cell states, clinical progression features, and functional phenotypes such as migration, invasion, and selected EMT-related marker changes, without positioning EMT as the sole mechanistic framework of the study.

Epigenetic dysregulation provides another plausible layer through which tumor-cell-state genes may be altered in PTC. Genome-wide DNA methylation analyses have identified CpG island methylator phenotype-related subclasses in PTC that are associated with advanced pathological stage, lymph node metastasis, chromosomal instability, and poorer clinical outcome (Gu et al., 2022). DNA methylation has also been explored as a translational biomarker source for distinguishing PTC from benign thyroid nodules, including plasma cell-free DNA methylation classifiers (Hong et al., 2023). Nevertheless, methylation changes observed in public datasets or after pharmacological DNA methyltransferase inhibition do not by themselves establish locus-specific causality. For CRABP1, it is therefore important to distinguish promoter-level methylation patterns, individual CpG-marker associations, pharmacological demethylation responses, and direct functional consequences of CRABP1 perturbation.

In this study, we integrated public single-cell RNA-sequencing datasets, CNV-based malignant-cell inference, TCGA transcriptomic and methylation data, and experimental validation models to investigate the role of CRABP1 in thyroid carcinoma. We first characterized a CRABP1-positive malignant epithelial population in PTC and evaluated its relationship with clinicopathological features, transcriptional programs, pathway enrichment, and inferred cell-cell communication. We then examined CRABP1 loss- and gain-of-function effects on migration, invasion, selected EMT-related gene expression, and xenograft growth, and assessed whether public methylation features and decitabine treatment were consistent with a methylation-associated regulatory model. This integrated framework was designed to define the cellular identity, clinical relevance, functional role, and candidate epigenetic regulation of CRABP1 while maintaining a clear boundary between association, pharmacological response, and locus-specific epigenetic causality.

## Materials and methods

### Bulk RNA-sequencing data acquisition

The external bulk RNA-seq dataset was used only for the CRABP1-high versus CRABP1-low exploratory GSEA shown in Supplementary Figure S2. It was derived from the previously published multicenter PTC cohort (Liu et al., 2025), which profiled tumor tissues from 157 patients with PTC, including 107 metastatic and 50 non-metastatic cases. This study did not generate that cohort or use it for the single-cell, TCGA, methylation, or functional analyses. The original publication states that its analyzed dataset is available from its corresponding author on reasonable request. In the present study, the dataset was used solely as an external transcriptomic context for pathway exploration and not for causal inference. The data sources, scopes, access conditions, and analytical uses are summarized in Supplementary Table S1.

### Single-cell RNA-sequencing data acquisition and sample selection

Publicly available human single-cell RNA-sequencing (scRNA-seq) datasets were obtained from the National Center for Biotechnology Information Gene Expression Omnibus (NCBI GEO). All papillary thyroid carcinoma (PTC) tumor samples available for scRNA-seq in three independent series were included, yielding 17 samples in total: GSE184362 (PTC1, PTC2, PTC3, PTC5, PTC8, PTC9, and PTC10; n = 7), GSE193581 (PTC1-PTC7; n = 7), and GSE250521 (PTC2-PTC4; n = 3). Adjacent normal tissues, metastatic lymph-node specimens, anaplastic thyroid carcinoma samples, and other non-PTC specimens present in the parent datasets were excluded. Sample information is shown in Table 1. Sample “PTC2” from the GSE193581 series was identified as having an ambiguous lymph node metastasis (LNM); therefore, this sample was excluded from the subsequent differential expression analyses to avoid potential confounding effects. Processed gene-by-cell count matrices were imported into R and analyzed using Seurat.

**TABLE 1:** Clinical characteristics and source of single-cell RNA-seq datasets used in this study.

Dataset	Sample	Stage	Dataset	Sample	Stage
GSE184362	PTC1	T4aN0M0	GSE193581	PTC3	T4aN1b
GSE184362	PTC2	T4aN1bM0	GSE193581	PTC4	T4aN1b
GSE184362	PTC3	T1bN1bM0	GSE193581	PTC5	T4aN1b
GSE184362	PTC5	T4aN1bM1	GSE193581	PTC6	T2aN1b
GSE184362	PTC8	T4aN1bM0	GSE193581	PTC7	T2N0
GSE184362	PTC9	T1bN1aM0	GSE250521	PTC2	N0
GSE184362	PTC10	T4aN1bM1	GSE250521	PTC3	N0
GSE193581	PTC1	T2N1b	GSE250521	PTC4	N1a
GSE193581	PTC2	T3bNx	​	​	​

### Quality control, normalization, and feature selection

A separate Seurat object was generated for each sample. Genes detected in fewer than 3 cells were discarded during object creation. Cells expressing more than 200 but fewer than 6,000 genes were retained. The fraction of mitochondrial transcripts was calculated from genes beginning with “MT”- as an additional quality-control metric.

Gene-expression values were normalized with Seurat’s LogNormalize procedure using a scale factor of 10,000. The 2,000 most variable genes were selected using the variance-stabilizing transformation (vst) method. Cell-cycle scores were calculated using the updated 2019 S-phase and G2/M-phase gene sets supplied with Seurat, and the S. Score and G2M.Score covariates were regressed out during data scaling.

### Batch correction, dimensionality reduction, and clustering

Principal-component analysis (PCA) was performed on the scaled expression matrix, retaining 30 principal components. To mitigate technical variation among samples and GEO series, Harmony integration was applied to the PCA embedding using sample identity as the batch covariate. The first 30 Harmony dimensions were used to construct the shared nearest-neighbor graph and to generate uniform manifold approximation and projection (UMAP) and t-distributed stochastic neighbor embedding (t-SNE) representations. Graph-based clustering was evaluated at resolutions of 0.3, 0.5, 0.7, and 0.9; a resolution of 0.5 was used for the final cell-cluster assignments and downstream analyses. Batch correction was assessed by visualizing the distribution and intermixing of cells from individual samples in the Harmony-derived UMAP space.

### Cell-type annotation

Cell clusters were manually annotated by integrating cluster-enriched genes with established lineage markers. Multiple concordant markers were considered for each assignment. Representative markers included CD79A, CD79B, and MS4A1 for B cells; JCHAIN, MZB1, IGKC, and IGHG1 for plasma cells; CD2, CD3D, and CD3E for T cells; NKG7 and KLRD1 for NK cells; C1QA, C1QB, C1QC, CD68, and FCN1 for macrophages; CSF3R, S100A8, and S100A9 for neutrophils; IDO1, CCR7, CD1E, and BATF3 for dendritic cells; PECAM1, RAMP2, VWF, and CLDN5 for endothelial cells; RGS5, PDGFRB, MYL9, and TAGLN for pericytes; COL1A1, COL1A2, DCN, and LUM for fibroblasts; and EPCAM, KRT18, KRT19, and CDH1 for epithelial cells. The CRABP1-positive malignant epithelial cell population was defined by retention of the epithelial marker program, enriched CRABP1 expression, and broad copy-number alterations consistent with the malignant epithelial compartment. The original analysis-object label was Epithelial_CRABP1; this label is referred to consistently as CRABP1-positive malignant epithelial cells in the text.

### InferCNV analysis

To infer copy number variations (CNVs) at the single-cell level, we applied the inferCNV R package to the epithelial cell populations. Macrophages were designated as the reference normal cell group, while ten epithelial subclusters (C0-C9) were defined as observation groups. Before inference, genes with a mean read count <1 across reference cells and genes expressed in fewer than three reference cells were excluded. A moving-average window of 101 genes was applied to smooth expression ratios, and the denoising option was enabled. Hierarchical clustering of cells was performed using Ward’s D2 linkage method (hclust_method = “ward.D2”). The hidden Markov model (HMM) was applied with the six-state model (HMM_type = “i6”; CNV states corresponding to 0, 0.5, 1, 1.5, 2, and >2) to predict discrete CNV states. CRABP1-positive malignant epithelial cells cells showing broad chromosomal copy-number alterations consistent with the malignant epithelial compartment were classified as CRABP1-positive malignant epithelial cells.

### Differential expression analysis between cell populations

Seurat’s FindAllMarkers function was applied using the default two-sided Wilcoxon rank-sum test in a one-versus-rest framework. Only positively enriched markers were returned, and genes were required to be detected in at least 15% of cells in either comparison group and to have an average log2 fold change of at least 0.15. Genes with a Bonferroni-adjusted P value < 0.05 and a positive average log2 fold change were considered significant cell-population markers. Significant marker sets were retained for downstream functional analyses.

### Functional enrichment analysis

Over-representation analysis was performed using clusterProfiler. Gene Ontology (GO) annotations were sourced from org. Hs.e.g.,.db (v3.18.0), and KEGG pathway annotations for *Homo sapiens* were retrieved via the KEGG REST API. Specifically, functional enrichments were calculated using enrichGO (covering all three GO sub-ontologies) and enrichKEGG, respectively. For GO enrichment, the background consisted of all human genes with available GO annotations in org. Hs.e.g.,.db. For KEGG enrichment, all human genes mapped to at least one KEGG pathway in the online KEGG database were used as the background. The enrichment plots display adjusted P values.

### Gene set enrichment analysis

Gene set enrichment analysis (GSEA) was performed using the Java-based GSEA software. All genes were ranked by log2 fold-change values derived from differential expression analysis with limma-voom. The ranked gene list was used as input for preranked GSEA against gene sets from the Molecular Signatures Database (MSigDB). For each gene set, the enrichment score (ES) was calculated, and 1,000 gene-set permutations were performed to generate the null distribution for statistical assessment. The normalized enrichment score (NES) was computed to account for differences in gene-set size. Because the legacy GSEA plot files available for this revision do not retain FDR q values, the Supplementary Figure S2 results are presented as exploratory pathway context and are not used as confirmatory evidence or to support causal claims. Full GSEA q-value reporting will require the archived ranked-gene input and GSEA result tables.

### Cell-cell communication analysis

Intercellular communication was computationally inferred using CellChat and the normalized RNA expression matrix. The complete human CellChatDB was used without restriction to a specific interaction category. Communication probabilities were calculated using the robust triMean estimator with population. size = TRUE, thereby incorporating the relative abundance of each cell population. Cell populations containing fewer than 10 cells were excluded with filterCommunication (min.cells = 10). Ligand-receptor and pathway-level communication probabilities were extracted using CellChat’s default significance threshold (P < 0.05), pathway-level communication probabilities were calculated with computeCommunProbPathway, and the resulting networks were aggregated with aggregateNet. These analyses generate hypothesis-level predictions; they do not demonstrate direct ligand-receptor binding or functional intercellular signaling.

### TCGA bulk RNA-sequencing differential expression analysis

Gene-level STAR-count data and corresponding clinical annotations for the TCGA Thyroid Carcinoma (TCGA-THCA) cohort were analyzed using DESeq2.

A DESeqDataSet was generated using a single-factor design. Size-factor estimation, dispersion estimation, and negative-binomial generalized linear modeling were performed using the standard DESeq workflow. The log2 fold-change coefficient was subsequently shrunk using the adaptive t prior implemented in apeglm. P values were adjusted for multiple comparisons with the Benjamini–Hochberg procedure. Differentially expressed genes were defined by an adjusted P value < 0.05 and an absolute shrunken log2 fold change > 1.

### Cell lines and culture conditions

The human thyroid cancer cell lines HTC-C3 (JCRB0164), ML-1 (DSMZ ACC 464), B-CPAP (DSMZ ACC 273), FTC-238 (ECACC 94060902), FTC-133 (ECACC 94060901), MB-1 (DSMZ ACC 638), and BHT-101 (DSMZ ACC 279) were maintained at 37 °C. HTC-C3 cells were cultured in high-glucose Dulbecco’s modified Eagle medium (DMEM; Gibco, 11965092) with 10% fetal bovine serum (FBS; Gibco, 10099141C). ML-1 cells were cultured in high-glucose DMEM with 10% heat-inactivated FBS, 2 mM L-glutamine, and 1 mM sodium pyruvate. B-CPAP and MB-1 cells were cultured in RPMI-1640 (Gibco, 11875093) containing 10% and 20% heat-inactivated FBS, respectively; the MB-1 medium also contained 2 mM L-glutamine. FTC-238 and FTC-133 cells were cultured in a 1:1 mixture of DMEM and Ham’s F-12 (Gibco, 11320033), 2 mM L-glutamine, and 5% or 10% FBS, respectively. BHT-101 cells were cultured in DMEM with 20% heat-inactivated FBS and 0.5% human serum. All cultures were maintained in a humidified atmosphere with 5% CO_2_ except BHT-101 cells, which were maintained with 10% CO_2_. Basal CRABP1 mRNA expression was measured by reverse-transcription quantitative PCR (RT-qPCR), and HTC-C3 and FTC-238 cells were selected as relatively high- and low-CRABP1 models, respectively. HTC-C3 is commonly used as an undifferentiated/anaplastic thyroid carcinoma-derived model, whereas FTC-238 is derived from metastatic follicular thyroid carcinoma. These complementary models were therefore used for thyroid carcinoma cell-line validation and are not intended to establish a PTC-specific cellular mechanism.

### CRABP1 knockdown and overexpression

HTC-C3 cells were transfected at approximately 60%–70% confluence with a non-targeting control siRNA (siCon; OriGene, SR30004) or one of three independent human CRABP1-targeting siRNAs (OriGene, SR300971). SiRNAs were used at 10 nM with Lipofectamine RNAiMAX (Invitrogen, 13778075) according to the manufacturer’s instructions. The medium was replaced after 18 h, and cells were collected 48 h after transfection. CRABP1 protein abundance was assessed by immunoblotting, and siCRABP1-3, which produced the strongest depletion, was used in subsequent experiments. FTC-238 cells were transfected with a Myc-DDK-tagged full-length human CRABP1 open reading frame in pCMV6-Entry (oeCRABP1; OriGene, RC223810) or the matched empty vector using Lipofectamine 3000 (Invitrogen, L3000015). CRABP1 overexpression was confirmed by immunoblotting before phenotypic analysis. The HTC-C3 cell preparations used for xenograft implantation are designated siCon and siCRABP1-3 in Figure 1.

**FIGURE 1:**
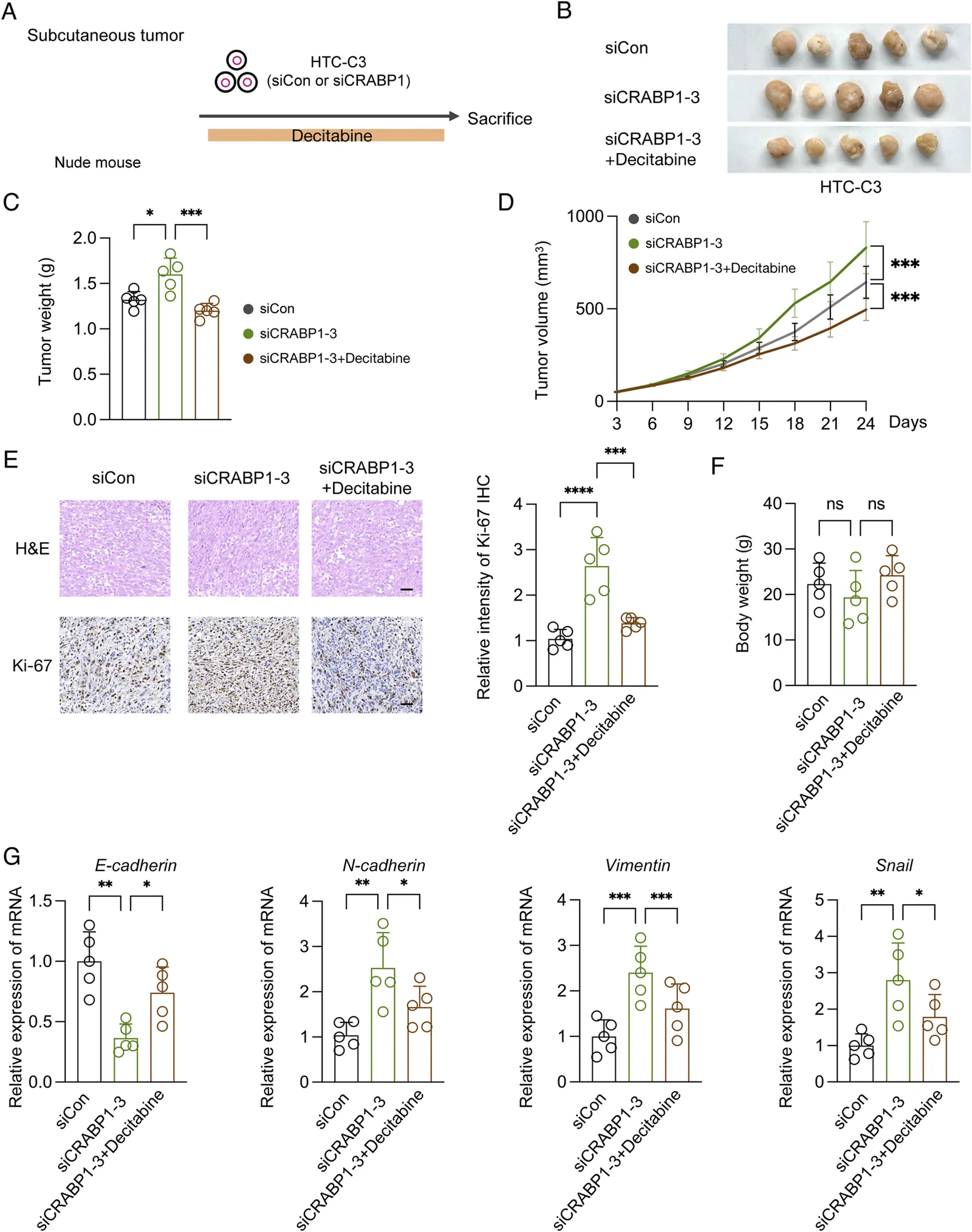
Decitabine is associated with attenuation of phenotypes in the CRABP1-depleted xenograft background. **(A)** Schematic of the subcutaneous xenograft experiment. Mice were assigned to siCon plus vehicle, siCRABP1-3 plus vehicle, or siCRABP1-3 plus decitabine. **(B)** Representative excised tumors at endpoint (n = 5 per group) **(C)** Terminal tumor weights. **(D)** Tumor-growth curves from day 3 to day 24. **(E)** Representative H&E− and Ki-67-stained xenograft sections. **(F)** Relative Ki-67 immunoreactivity and terminal mouse body weights. **(G)** RT-qPCR analysis of EMT-related transcripts in xenograft tissues. As the design lacks a siCon plus decitabine group, it cannot separate a general drug effect from a CRABP1-dependent interaction or establish a CRABP1-specific epigenetic rescue.

### Decitabine treatment

For *in vitro* experiments, decitabine (5-aza-2′-deoxycytidine; Selleck Chemicals, S1200) was dissolved in dimethyl sulfoxide and applied at 5 μM for 5 days. Fresh decitabine-containing medium was prepared and replaced every 24 h; control cells received an equal volume of vehicle. Decitabine or vehicle was combined factorially with CRABP1 depletion in HTC-C3 cells and CRABP1 overexpression in FTC-238 cells.

### RNA extraction and RT-qPCR

Total RNA was extracted with TRIzol reagent (Invitrogen, 15596018). One microgram of total RNA was reverse transcribed using the RevertAid First Strand cDNA Synthesis Kit (Thermo Fisher Scientific, K1622). Quantitative PCR was performed in 20-μL reactions using PowerUp SYBR Green Master Mix (Applied Biosystems, A25742). Cycling conditions were 50 °C for 2 min and 95 °C for 2 min, followed by 40 cycles of 95 °C for 15 s and 60 °C for 1 min and subsequent melt-curve analysis. Each biological sample was analyzed in technical triplicate. Relative mRNA abundance was calculated by the 2^^−ΔΔ^Ct method using GAPDH as the reference gene. Cell experiments comprised three independent biological replicates per group, and xenograft-tissue analyses comprised five tumors per group.

Primer sequences (5’ - 3′) were as follows: CRABP1, forward CAG​GAC​GGG​GAT​CAG​TTC​TA and reverse CGC​CAA​ACG​TCA​GGA​TAA​GT; E-cadherin, forward CGA​GAG​CTA​CAC​GTT​CAC​GG and reverse GGG​TGT​CGA​GGG​AAA​AAT​AGG; N-cadherin, forward TCA​GGC​GTC​TGT​AGA​GGC​TT and reverse ATG​CAC​ATC​CTT​CGA​TAA​GAC​TG; Vimentin, forward GAC​GCC​ATC​AAC​ACC​GAG​TT and reverse CTT​TGT​CGT​TGG​TTA​GCT​GGT; Snai1, forward GCT​CCA​CAA​GCA​CCA​AGA​GT and reverse ATT​CCA​TGG​CAG​TGA​GAA​GG; and GAPDH, forward GGA​GCG​AGA​TCC​CTC​CAA​AAT and reverse GGC​TGT​TGT​CAT​ACT​TCT​CAT​GG.

### Western blotting

Cells were lysed in RIPA buffer containing protease inhibitors, and protein concentration was determined using a bicinchoninic acid assay. Equal amounts of protein (30 μg per lane) were separated by 12% SDS-PAGE and transferred to polyvinylidene difluoride membranes. Membranes were blocked in 5% bovine serum albumin in Tris-buffered saline containing 0.1% Tween 20 and incubated overnight at 4 °C with mouse monoclonal anti-CRABP1 (clone C-1; Sigma-Aldrich, C1608; RRID: AB_258751; 1:1,000) or rabbit polyclonal anti-β-tubulin (Abcam, ab6046; RRID: AB_2210370; 1:4,500). Membranes were then incubated for 1 h at room temperature with horse anti-mouse IgG-HRP (Cell Signaling Technology, 7076; RRID: AB_330924; 1:2,000) or goat anti-rabbit IgG-HRP (Abcam, ab6721; RRID: AB_955447; 1:6,000). Signals were detected by enhanced chemiluminescence and quantified using ImageJ; CRABP1 band intensity was normalized to β-tubulin.

### Transwell migration and invasion assays

Migration and invasion were evaluated using 24-well Transwell inserts with 6.5-mm, 8.0-μm-pore polycarbonate membranes (Corning, 3422). Migration inserts were left uncoated. For invasion assays, the upper membrane was coated with 100 μL of 2% Matrigel matrix (Corning, 356,234). Cells were suspended in serum-free medium and seeded into the upper chamber at 5 × 10^4^ cells per insert for migration or 3 × 10^4^ cells per insert for invasion; medium containing 10% FBS was added to the lower chamber. After 24 h at 37 °C, non-migrated cells were removed from the upper membrane surface. Cells on the lower surface were fixed in anhydrous methanol for 30 min and stained with 0.2% crystal violet (Sigma-Aldrich, V5265). Six prespecified fields per insert were imaged at 20×. Stained cells were counted using ImageJ, averaged for each insert, and expressed relative to the matched control. Three independently performed experiments were analyzed per group.

### Vimentin immunofluorescence

Cells grown on glass coverslips were fixed in 4% paraformaldehyde for 15 min, permeabilized with 0.1% Triton X-100 for 30 min, and blocked with 1% bovine serum albumin for 15 min. Cells were incubated overnight at 4 °C with rabbit monoclonal anti-Vimentin (clone D21H3; Cell Signaling Technology, 5741; RRID: AB_10695459; 1:200), followed by Alexa Fluor 488-conjugated goat anti-rabbit IgG (Invitrogen, A-11008; RRID: AB_143165; 1:500) for 1 h at room temperature in the dark. Nuclei were counterstained with DAPI (Invitrogen, D1306; 300 nM) for 5 min. All images were acquired using identical exposure and acquisition settings. Vimentin fluorescence intensity was quantified using ImageJ and normalized to the matched control. Three independently prepared coverslips were analyzed per group.

### Public methylation and expression analyses

CRABP1 promoter-level methylation was examined in the UALCAN portal using TCGA-THCA Illumina HumanMethylation450 data (accessed 22 July 2026). UALCAN aggregates β-values for probes annotated to TSS200 and TSS1500, located within 1,500 bp upstream of the transcription start site. The displayed analyses included normal thyroid tissue (n = 56), primary thyroid carcinoma (n = 507), N0 tumors (n = 231), N1 tumors (n = 226), and the indicated histological categories.

Probe-level methylation across the CRABP1 locus and its relationship with CRABP1 expression were visualized in MEXPRESS using TCGA-THCA data (accessed 22 July 2026). The CRABP1 position profile was independently examined in OncoDB 2.0, which compared mean probe β-values between THCA tumors (n = 505) and normal thyroid tissues (n = 50). Illumina HumanMethylation450 annotation assigns cg02276974 to the CRABP1 gene body and the south shore of a CpG island; mapping to the CRABP1 MANE Select transcript places it in intron 2 (GRCh37/hg19 chr15:78,634,235; GRCh38/hg38 chr15:78,341,893).

Sample-level cg02276974 β-values and CRABP1 RNA-sequencing abundance [log_2_ (normalized count +1)] were obtained from the UCSC Xena TCGA-THCA hub. Primary tumors with complete methylation and expression measurements were matched by TCGA sample barcode (n = 505). Pearson correlation and ordinary least-squares regression were used to quantify the displayed association. The interpretation boundaries among the aggregate promoter, probe-level, locus-profile, and matched-sample measurements are summarized in Supplementary Table S2.

### Subcutaneous xenograft experiment

Seven-week-old female BALB/c nude mice were acclimatized for 1 week under specific-pathogen-free conditions. HTC-C3 cells designated siCon or siCRABP1-3 (1 × 10^7^ cells in 0.1 mL of a 1:1 mixture of serum-free medium and Matrigel) were injected subcutaneously into the right flank. Mice were randomized into three groups (n = 5 per group): siCon plus vehicle, siCRABP1-3 plus vehicle, and siCRABP1-3 plus decitabine. Decitabine (Sigma-Aldrich, A3656) was administered intraperitoneally at 5 mg/kg once weekly on days 2, 9, 16, and 23; mice in the two vehicle groups received volume-matched sterile saline. Tumor length and width were measured with calipers on days 3, 6, 9, 12, 15, 18, 21, and 24, and tumor volume was calculated as length × width^2^/2. Body weight was recorded concurrently. On day 24, mice were euthanized, and tumors were excised, weighed, photographed, and processed for histology and RNA analysis.

### Histology and Ki-67 immunohistochemistry

Excised tumors were fixed in 4% paraformaldehyde, paraffin embedded, and sectioned at 4–5 μm. Sections were stained with hematoxylin and eosin or processed for Ki-67 immunohistochemistry. After deparaffinization and rehydration, heat-mediated antigen retrieval was performed in sodium citrate buffer (pH 6.0), and endogenous peroxidase activity was blocked with 3% H_2_O_2_. Sections were incubated overnight at 4 °C with rabbit polyclonal anti-Ki-67 (Proteintech, 27309-1-AP; RRID: AB_2756525; 1:5,000), followed by HRP-conjugated goat anti-rabbit IgG (Abcam, ab7090; RRID: AB_955417; 1:2,000) for 50 min at room temperature. Signal was developed with 3,3′-diaminobenzidine, and nuclei were counterstained with hematoxylin. Five prespecified viable-tumor fields per mouse were quantified and averaged, with the mouse treated as the experimental unit.

### Statistical analysis

Statistical analyses were performed using GraphPad Prism version 10.2.3, and data are presented as mean ± SD. Two-group cell experiments were analyzed using two-tailed unpaired Student's t-tests. A two-way ANOVA was fitted with decitabine treatment and CRABP1 manipulation as factors, followed by Sidak’s multiple-comparisons test. Terminal xenograft endpoints were analyzed separately using ordinary one-way ANOVA followed by Tukey’s multiple-comparisons test. Serial tumor volumes were analyzed using repeated-measures two-way ANOVA followed by Sidak’s multiple-comparisons test for the indicated day-24 comparisons. Pearson correlation and ordinary least-squares regression were used for the TCGA methylation-expression analysis. RT-qPCR inferential analyses were performed on ΔCt values, whereas 2^−ΔΔCt values are displayed. Differential-expression P values were adjusted by Bonferroni correction for single-cell markers and by the Benjamini–Hochberg procedure for TCGA bulk RNA-seq. Enrichment plots display adjusted P values; the exact enrichment adjustment procedure requires confirmation from the archived clusterProfiler output. All tests were two-sided, and P < 0.05 was considered statistically significant unless an adjusted P value is specified.

## Results

### Integrated single-cell and CNV analyses identify a CRABP1-positive malignant epithelial population in papillary thyroid carcinoma

To define the cellular context of CRABP1 expression in papillary thyroid carcinoma (PTC), we integrated publicly available single-cell RNA-sequencing datasets from 17 tumor samples. After quality control, normalization, dimensionality reduction, and Harmony-based batch correction, cells from different samples were broadly intermingled in the UMAP space, supporting effective mitigation of sample-associated technical variation. Canonical lineage markers resolved the major tumor microenvironment populations, including epithelial cells, fibroblasts, endothelial cells, pericytes, macrophages, neutrophils, dendritic cells, T cells, NK cells, B cells, and plasma cells (Figures 2A–C).

**FIGURE 2:**
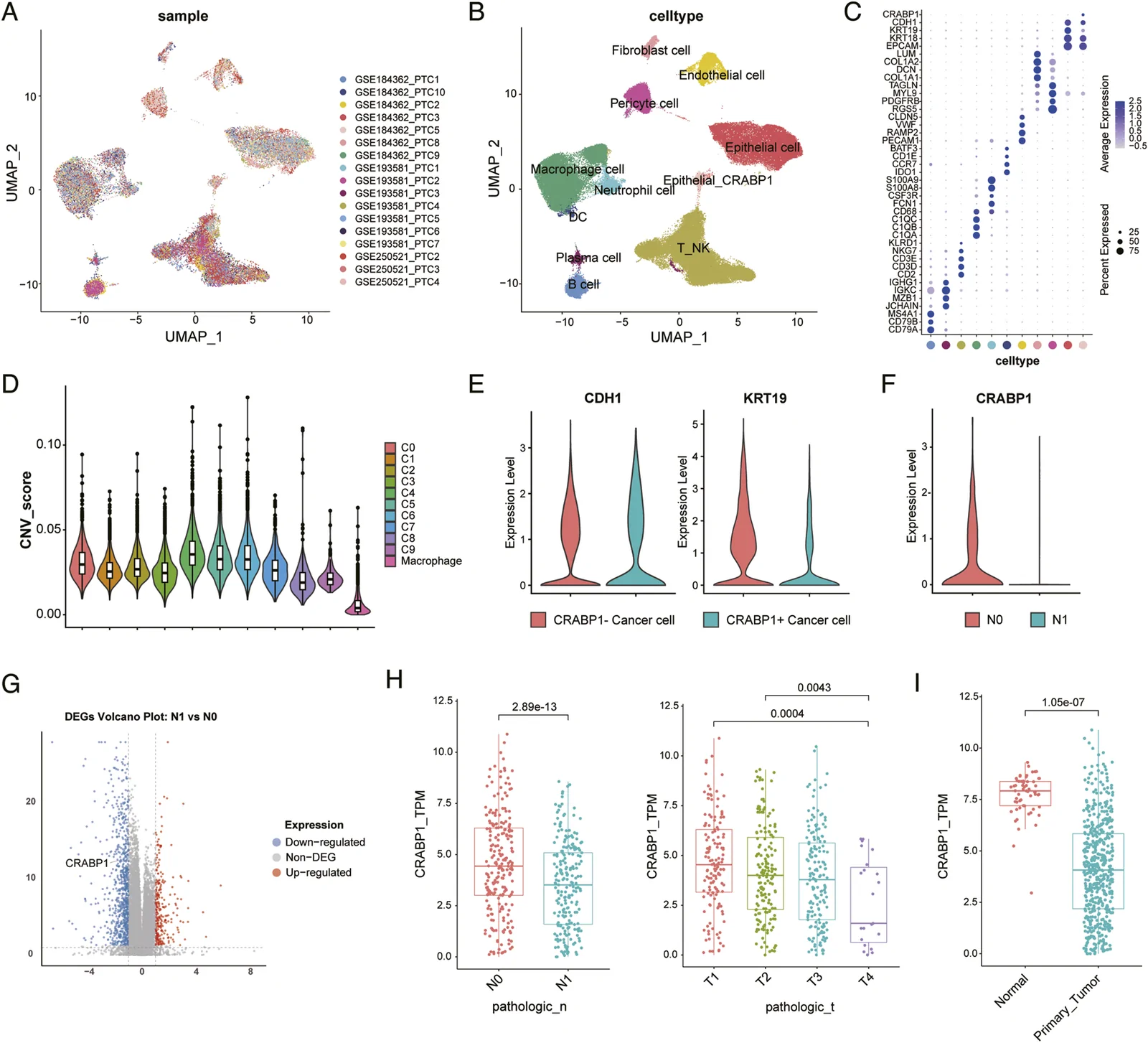
Single-cell identification of CRABP1-positive malignant epithelial cells and observational clinical associations in thyroid carcinoma **(A)** Uniform manifold approximation and projection (UMAP) of the integrated single-cell transcriptomic dataset, with cells colored by sample of origin. The extensive intermixing of cells from different samples indicates mitigation of sample-associated batch effects **(B)** UMAP visualization of the major cell populations identified by canonical lineage markers **(C)** Dot plot showing the expression of representative canonical marker genes across the annotated cell populations. Dot size represents the percentage of cells expressing each gene, whereas color intensity indicates the scaled average expression level **(D)** Violin plots depicting the distribution of CNV scores across the ten epithelial subclusters (C0-C9), as inferred by the InferCNV pipeline. Each dot represents an individual cell. Subcluster C8 displayed the lowest CNV score and was used as the normal epithelial reference population **(E)** Violin plots showing CDH1 and KRT19 expression in CRABP1-negative and CRABP1-positive malignant epithelial cells **(F)** Violin plot comparing CRABP1 expression in CRABP1-positive malignant epithelial cells from N0 and N1 tumors **(G)** Volcano plot of differentially expressed genes in N1 versus N0 tumors in TCGA-THCA bulk RNA-sequencing data **(H)** Boxplots with individual samples showing CRABP1 transcript abundance by pathological N status and T category **(I)** Boxplot with individual samples comparing CRABP1 transcript abundance in adjacent normal thyroid tissues and primary tumors. Panels F–I are observational associations and do not demonstrate causal clinical effects.

To further dissect the heterogeneity within the epithelial compartment, we subclustered the epithelial cells into ten distinct subclusters (C0–C9) via unsupervised clustering (Supplementary Figure S1A). Copy number variation (CNV) was inferred for each individual epithelial cell using the InferCNV pipeline, with macrophages serving as the reference (normal) population. Based on the resulting CNV scores and CRABP1 expression levels, the epithelial cells were classified into three categories: non-malignant epithelial cells, CRABP1-positive malignant epithelial cells, and CRABP1-negative malignant epithelial cells (Figure 2D; Supplementary Figure S1B,C). Subcluster C8 displayed the lowest CNV score among all epithelial groups and was designated as the normal epithelial population for subsequent analyses. CRABP1-positive population (subcluster C6) displayed comparably high CNV levels that were similar to those of other malignant populations (Figure 2D), confirming its malignant nature. Importantly, this CRABP1-positive malignant population retained expression of typical epithelial markers such as CDH1 and KRT19 (Figure 2E), indicating that CRABP1 expression marked a distinct epithelial-state subset rather than a non-epithelial contaminating population.

### Reduced CRABP1 expression is associated with more advanced clinicopathological features in TCGA-THCA

We next examined whether CRABP1 expression was associated with clinical features in thyroid carcinoma. In the single-cell dataset, CRABP1 expression in CRABP1-positive malignant epithelial cells differed between tumors from patients without lymph-node metastasis (N0) and those with lymph-node metastasis (N1) (Figure 2F). In the TCGA-THCA bulk RNA-sequencing cohort, differential-expression analysis comparing N1 with N0 tumors identified CRABP1 among the genes associated with nodal status (Figure 2G). Boxplot analysis further showed lower CRABP1 transcript abundance in N1 tumors than in N0 tumors and variation across pathological T categories (Figure 2H). CRABP1 expression was also lower in primary thyroid tumors than in adjacent normal thyroid tissues (Figure 2I). These unadjusted observational analyses support an association between lower CRABP1 expression and progression-associated clinicopathological features; they do not establish that CRABP1 causes clinical progression. In light of the CNV-based malignant-cell annotation, the CRABP1-positive malignant epithelial state may represent a transcriptional program that is less prevalent or less transcriptionally prominent in more advanced disease contexts. To further characterize pathway-level differences associated with CRABP1 expression in the external bulk cohort, exploratory GSEA showed lower focal-adhesion and tight-junction enrichment and higher oxidative-phosphorylation and ATP-synthesis-coupled-electron-transport enrichment in the CRABP1-low group (Supplementary Figures S2A–D).

### CRABP1-positive malignant epithelial cells show distinct transcriptional, pathway, epigenetic, and inferred communication features.

To explore regulatory and functional features of the CRABP1-positive malignant epithelial state, we evaluated selected CpG-region methylation, marker expression, pathway enrichment, and computationally inferred cell-cell communication. TCGA-THCA methylation analysis showed N-stage-associated differences in beta values at three CRABP1-associated CpG regions (Figure 3A). These region-level comparisons are analytically distinct from the aggregate promoter-probe analysis and the cg02276974-focused sample-matched correlation described below. CLDN1 and ID1 showed different expression distributions between conventional epithelial cells and CRABP1-positive malignant epithelial cells (Figures 3B,C). Genes downregulated or upregulated in CRABP1-positive malignant epithelial cells were enriched in distinct KEGG pathways and Gene Ontology biological processes, indicating transcriptional differences between epithelial-cell states (Figures 3D–G). CellChat predicted APP-CD74 and PPIA-BSG ligand-receptor networks between CRABP1-positive malignant epithelial cells and selected macrophage subpopulations (Figures 3H,I). These computational predictions nominate candidate communication axes for future experimental testing; they do not establish direct signaling between these cell populations.

**FIGURE 3:**
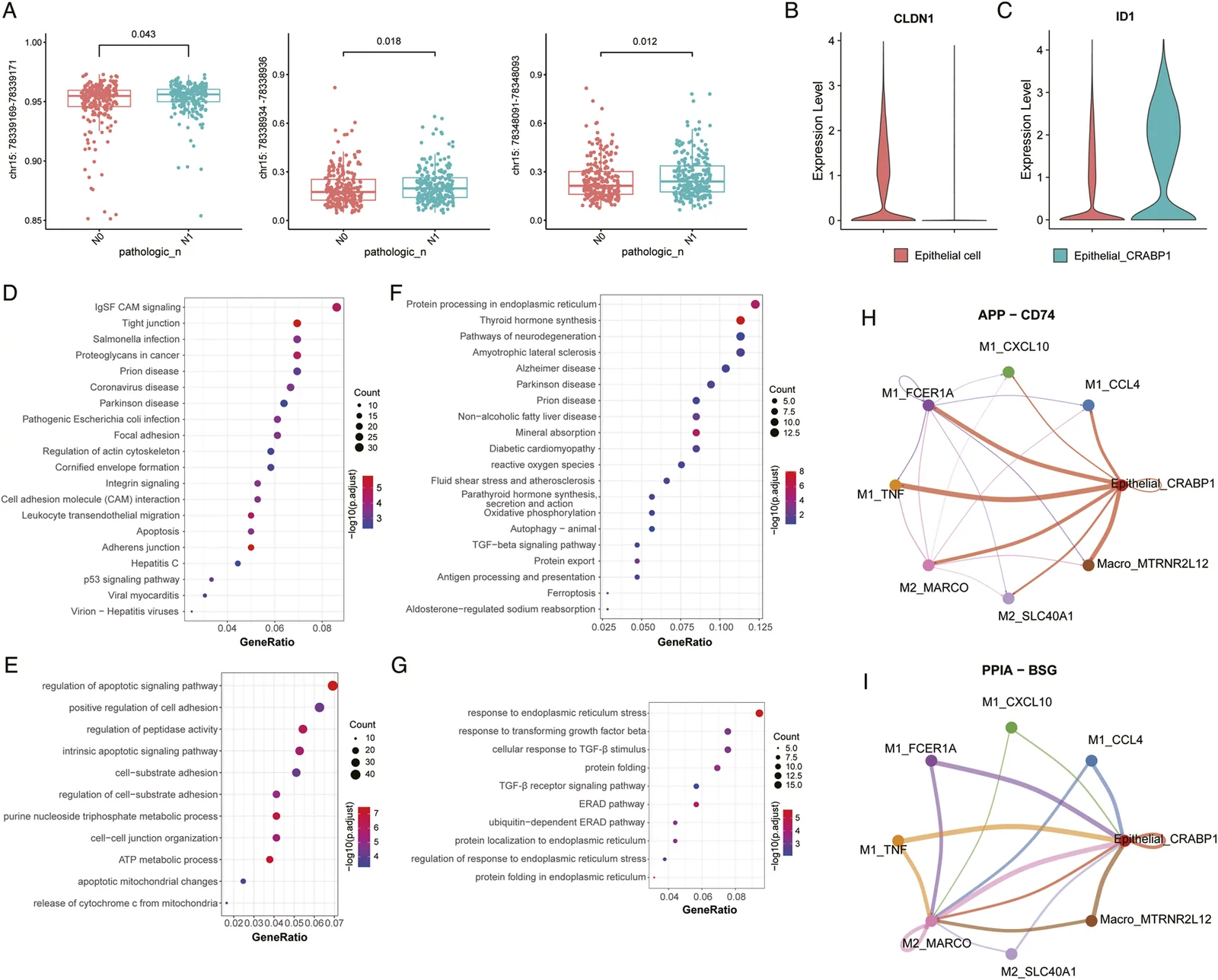
Transcriptional, methylation-region, and computationally inferred communication features of CRABP1-positive malignant epithelial cells **(A)** DNA methylation β values at three CRABP1-associated CpG regions in N0 and N1 tumors from TCGA-THCA. These region-level comparisons are distinct from the promoter aggregate and cg02276974 analyses in **(B,C)** Violin plots showing CLDN1 and ID1 expression in conventional epithelial cells and CRABP1-positive malignant epithelial cells. **(D–G)** KEGG and Gene Ontology enrichment analyses of downregulated and upregulated genes in CRABP1-positive malignant epithelial cells. The horizontal axis represents gene ratio, dot size indicates gene count, and color represents -log10 adjusted P value **(H,I)** CellChat-predicted ligand-receptor communication networks between CRABP1-positive malignant epithelial cells and indicated macrophage subpopulations for APP-CD74 and PPIA-BSG. Edge width represents inferred communication probability. These panels are computational predictions and hypothesis-generating only; they do not establish direct signaling.

### CRABP1 restrains migration, invasion, and EMT-related transcriptional changes in thyroid cancer cells

Among seven thyroid cancer cell lines, CRABP1 mRNA abundance was highest in HTC-C3 cells and comparatively low in FTC-238 cells, which were therefore selected as complementary loss- and gain-of-function thyroid carcinoma models, respectively. In HTC-C3 cells, siCRABP1-3 produced the strongest reduction in CRABP1 protein among the three tested siRNAs and was selected for subsequent assays. In FTC-238 cells, CRABP1 overexpression was confirmed by immunoblotting (Figures 4A–C). Functionally, CRABP1 depletion increased migration and invasion in HTC-C3 cells, whereas CRABP1 overexpression reduced both endpoints in FTC-238 cells (Figures 4D,E). CRABP1 knockdown decreased E-cadherin and increased N-cadherin, Vimentin, and Snail mRNA expression, whereas CRABP1 overexpression produced the reciprocal expression pattern (Figures 4F,G). These results support an association between CRABP1 manipulation and migration, invasion, and EMT-related transcriptional changes in thyroid carcinoma cell lines; because the two models do not recapitulate PTC specifically, they are interpreted as complementary to the PTC single-cell and TCGA analyses rather than as direct PTC-specific mechanistic evidence.

**FIGURE 4:**
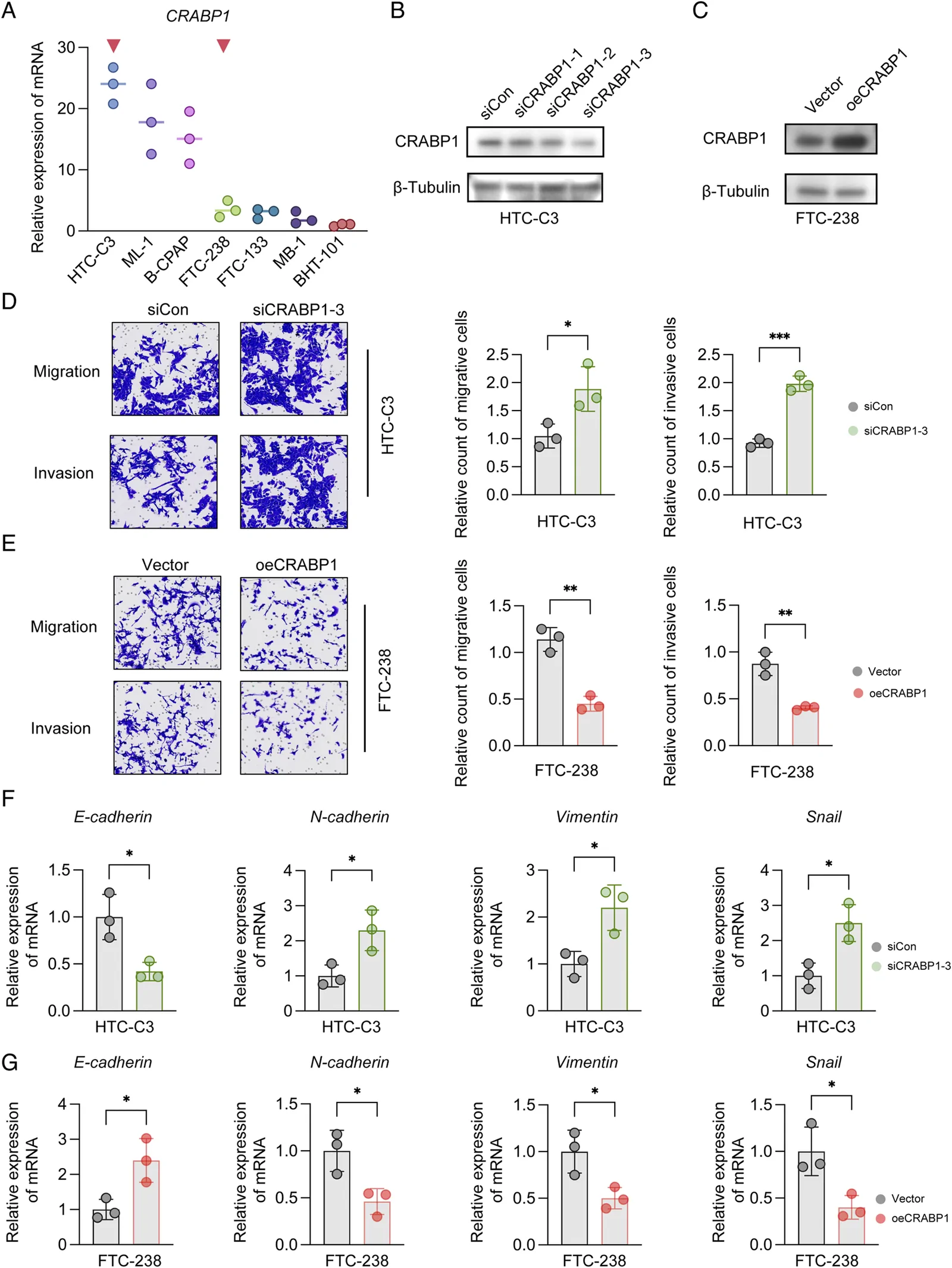
CRABP1 manipulation is associated with migration, invasion, and EMT-related transcriptional changes in complementary thyroid carcinoma cell-line models. **(A)** RT-qPCR analysis of relative CRABP1 mRNA expression in seven thyroid cancer cell lines. HTC-C3 and FTC-238 were selected as relatively high- and low-CRABP1 models, respectively. **(B)** Immunoblot analysis of CRABP1 in HTC-C3 cells transfected with control siRNA or three CRABP1-targeting siRNAs. **(C)** Immunoblot analysis of CRABP1 in FTC-238 cells transfected with empty vector or oeCRABP1. β-Tubulin served as the loading control. **(D,E)** Representative Transwell images and relative numbers of migrated and invaded cells after CRABP1 depletion in HTC-C3 cells or CRABP1 overexpression in FTC-238 cells **(F,G)** RT-qPCR analysis of EMT-related transcripts. HTC-C3 and FTC-238 represent undifferentiated/anaplastic and metastatic follicular thyroid carcinoma contexts, respectively; these data provide broader thyroid carcinoma validation and do not by themselves establish PTC-specific mechanisms.

### Public methylation analyses distinguish promoter-region changes from a weak intronic/gene-body cg02276974 expression association

We then evaluated whether distinct public DNA methylation measurements were associated with CRABP1 expression in thyroid carcinoma. UALCAN analysis of TCGA-THCA HumanMethylation450 data showed higher aggregate methylation of CRABP1 TSS200/TSS1500 promoter probes in primary tumors than in normal thyroid tissues. Increased promoter-level methylation was also observed in both N0 and N1 tumors relative to normal tissues, with variation across histological categories (Figures 5A–C). These aggregate promoter-probe estimates are distinct from locus-wide MEXPRESS probe visualization and the cg02276974 analysis. MEXPRESS showed heterogeneous probe-wise methylation-expression relationships across the CRABP1 locus (Figure 5D). OncoDB position profiling showed higher mean cg02276974 beta values in THCA tumors than in normal thyroid tissues. Annotation places cg02276974 in the CRABP1 gene body and CpG-island south shore, and transcript mapping places it in intron 2 rather than within the TSS200/TSS1500 promoter-probe region (Figure 5E). In 505 sample-matched primary THCA tumors, cg02276974 methylation was weakly inversely associated with CRABP1 RNA-sequencing expression (Pearson r = -0.2069, R^2^ = 0.0428; P < 0.001; Figure 5F). Thus, cg02276974 explains only a small fraction of expression variation and is reported as an intronic/gene-body association marker, not as a validated cis-regulatory methylation site or causal silencing element.

**FIGURE 5:**
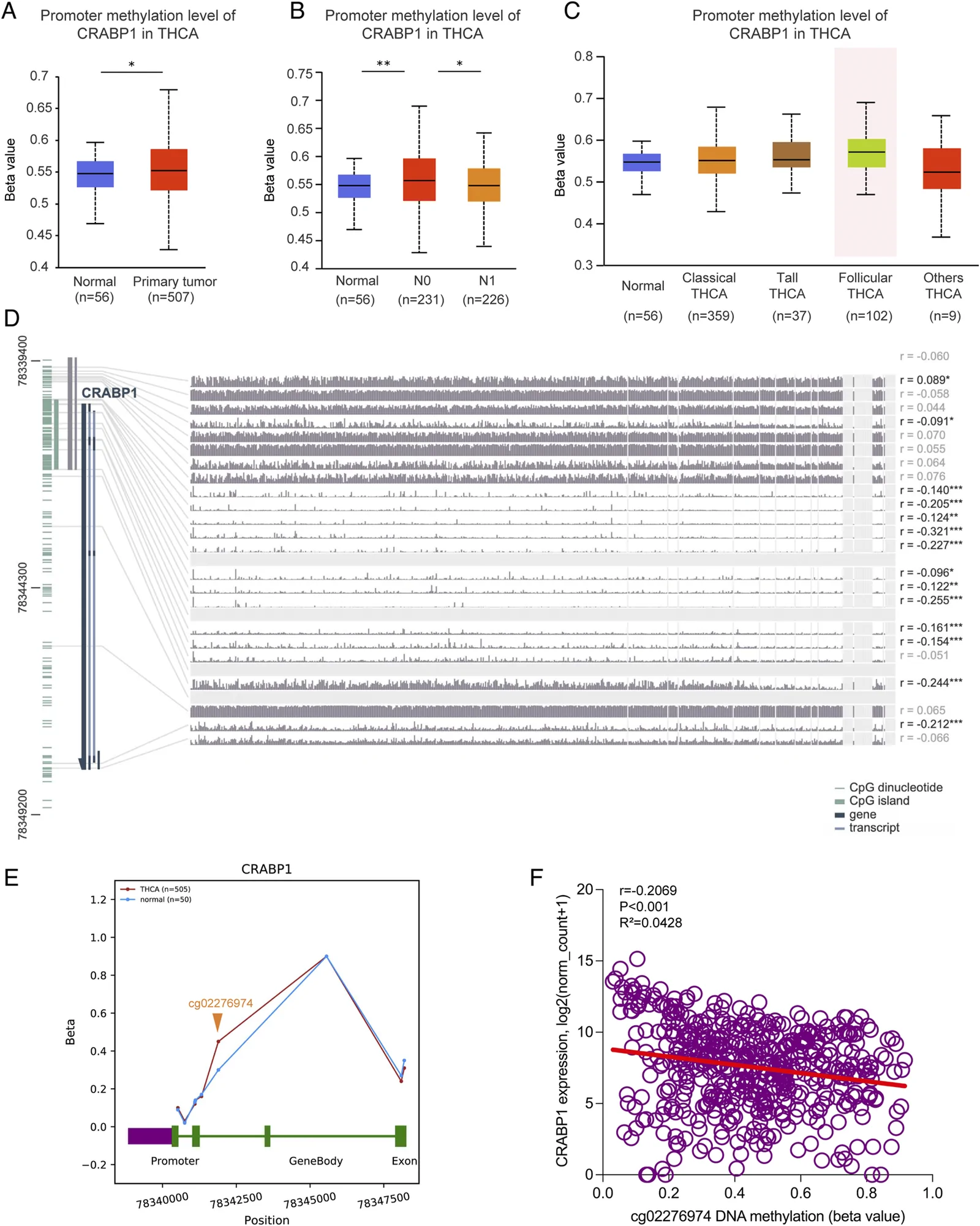
Distinct public methylation analyses show promoter-region differences and a weak cg02276974 intronic/gene-body expression association. **(A–C)** UALCAN aggregate methylation of CRABP1 TSS200/TSS1500 promoter probes in TCGA-THCA, comparing normal thyroid tissues with primary tumors, N0 and N1 tumors, and histological categories. **(D)** MEXPRESS visualization of probe-level methylation across the CRABP1 locus and probe-wise expression correlations. **(E)** OncoDB position profile of CRABP1 methylation in THCA tumors and normal thyroid tissues, with cg02276974 highlighted. The probe is annotated to the CRABP1 gene body and CpG-island south shore and maps to intron 2. **(F)** Correlation between cg02276974 methylation beta values and CRABP1 RNA-sequencing expression in 505 sample-matched primary THCA tumors. The fitted linear regression has Pearson r = −0.2069, P < 0.001, and R^2^ = 0.0428. The UALCAN, MEXPRESS, OncoDB, and cg02276974 analyses quantify different features and should not be treated as equivalent methylation measurements. Cg02276974 is an association marker, not a validated regulatory site.

### Decitabine pharmacologically alters migratory, invasive, and vimentin-associated phenotypes in CRABP1-manipulated thyroid carcinoma cells

After identifying methylation-associated alterations in public datasets, we tested whether pharmacological DNA methyltransferase inhibition altered cellular phenotypes in CRABP1-manipulated models. In HTC-C3 cells, decitabine reduced migrated and invaded cell numbers in both the siCon and siCRABP1-3 backgrounds. In FTC-238 cells, decitabine also reduced migration and invasion in both the empty-vector and oeCRABP1 backgrounds, with the lowest mean values in the oeCRABP1 plus decitabine group (Figures 6A,B). Vimentin immunofluorescence followed a similar pharmacological pattern within each genetic background (Figure 6C). Because decitabine has genome-wide effects, these findings are interpreted as drug responses in a methylation-associated context. They neither isolate CRABP1-specific mediation nor establish cg02276974-specific methylation regulation.

**FIGURE 6:**
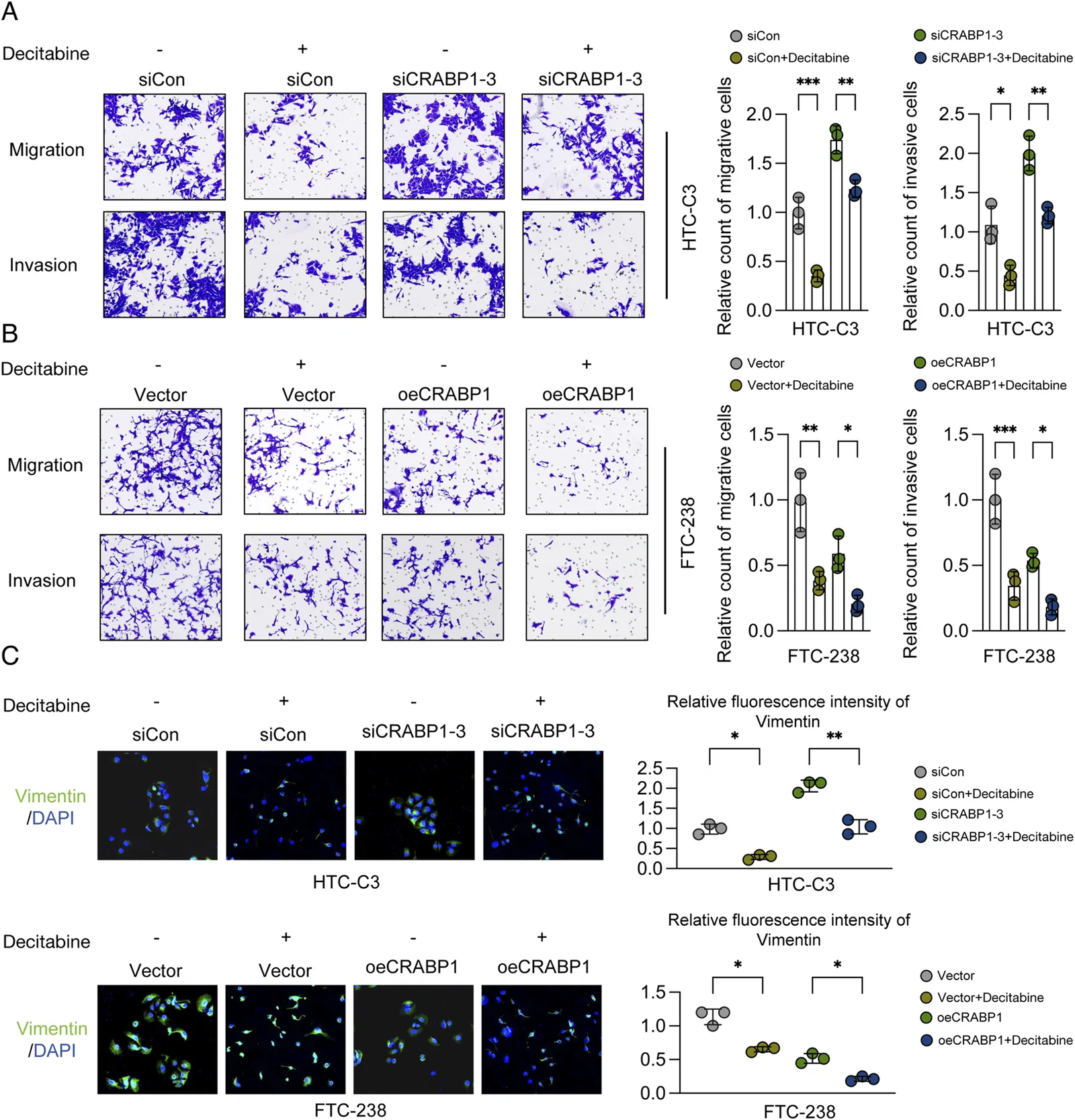
Decitabine pharmacologically alters migration, invasion, and Vimentin-associated phenotypes in CRABP1-manipulated thyroid carcinoma cells. **(A)** Representative Transwell images and relative migrated and invaded cell numbers in HTC-C3 cells after siCon or siCRABP1-3 transfection, with or without decitabine. **(B)** Corresponding FTC-238 experiments after empty-vector or oeCRABP1 transfection, with or without decitabine. **(C)** Representative Vimentin immunofluorescence images and relative Vimentin fluorescence intensity. Vimentin is green and nuclei are DAPI-stained blue. Because decitabine has genome-wide effects, these comparisons show pharmacological responses in a methylation-associated context and do not demonstrate CRABP1-specific or cg02276974-specific rescue.

### Decitabine is associated with attenuation of phenotypes in the CRABP1-depleted xenograft background

In the subcutaneous xenograft model, CRABP1-depleted tumors showed greater terminal tumor weight and a higher tumor-volume trajectory than control tumors during the observation period (Figures 1A–D). Within the CRABP1-depleted background, decitabine treatment was associated with lower terminal tumor weight and tumor volume. Ki-67 immunoreactivity increased after CRABP1 depletion and was lower in the decitabine-treated CRABP1-depleted group, whereas terminal body weight did not differ significantly among groups (Figures 1E,F). RT-qPCR analysis of xenograft tissues showed lower E-cadherin and higher N-cadherin, Vimentin, and Snail expression after CRABP1 depletion; marker expression differed in the decitabine-treated CRABP1-depleted group (Figure 1G). Because this *in vivo* design did not include a siCon plus decitabine group, it cannot separate a general decitabine effect from a CRABP1-dependent pharmacological interaction. These findings are therefore reported as attenuation within the CRABP1-depleted background, not as CRABP1-specific or locus-specific rescue.

## Discussion

Papillary thyroid carcinoma (PTC) is characterized by substantial intertumoral and intratumoral heterogeneity, yet the malignant epithelial states associated with progression remain incompletely defined. Previous studies have reported reduced CRABP1 expression in thyroid cancer and linked CRABP1 loss to invasive phenotypes (Hawthorn et al., 2004; Hsu et al., 2023). However, the cellular context of CRABP1 expression within the PTC tumor ecosystem and its relationship with epigenetic regulation have not been systematically integrated. The present study addresses this gap by combining single-cell transcriptomic analysis, CNV-based malignant-cell identification, public TCGA transcriptomic and methylation analyses, and experimental validation in thyroid cancer models.

A central finding of this study is the identification of a CRABP1-positive malignant epithelial population in PTC. This result refines CRABP1 from a bulk tumor-associated gene into a marker of a defined malignant epithelial state. CNV analysis further supports the malignant identity of this population, strengthening the interpretation that CRABP1 expression reflects tumor-cell heterogeneity rather than only variation among non-malignant epithelial compartments. This cellular-state perspective is consistent with recent single-cell studies showing that PTC progression involves diverse malignant thyrocyte programs, altered differentiation states, and coordinated remodeling of the tumor microenvironment (Pu et al., 2021; Yan et al., 2021; Wang et al., 2026). Within this landscape, the CRABP1-positive malignant epithelial state represents a distinct component of PTC heterogeneity with clinicopathological and functional relevance.

The clinical and experimental results support an invasion-associated suppressive role of CRABP1 in thyroid carcinoma. CRABP1 expression was lower in tumors with lymph-node metastasis in single-cell and TCGA-based observational analyses, but these associations do not establish a direct effect on clinical progression. In complementary thyroid carcinoma cell-line models, CRABP1 knockdown increased migration and invasion, whereas CRABP1 overexpression reduced these phenotypes. *In vivo*, CRABP1 depletion was associated with increased xenograft growth and EMT-related marker changes. These observations extend prior reports of CRABP1 downregulation and 5-prime-region hypermethylation in PTC(Huang et al., 2003) and CRABP1 loss-associated migration, invasion, and mesenchymal marker changes in thyroid cancer models (Hsu et al., 2023). The cell-line findings should be interpreted in a broader thyroid carcinoma context, complementary to rather than definitive for PTC-specific biology.

Another contribution of this work is the integration of distinct public CRABP1 methylation measurements with malignant-cell state and functional phenotype. Earlier work described CRABP1 5-prime-region hypermethylation in PTC(Huang et al., 2003), and broader methylation-based studies have linked epigenetic subtypes and methylation markers to PTC progression and diagnosis (Gu et al., 2022; Hong et al., 2023). Here, aggregate promoter-probe methylation, N-stage-associated CpG-region differences, and the weak intronic/gene-body cg02276974 expression association are reported as separate observations rather than equivalent measurements. Decitabine altered migration, invasion, and Vimentin-associated phenotypes across CRABP1-manipulated models. Given its genome-wide pharmacology and the absence of a decitabine-only *in vivo* group, these experiments support a methylation-associated pharmacological context but do not demonstrate CRABP1-specific, cg02276974-specific, or locus-specific epigenetic causality.

The inferred communication analysis provides a hypothesis-generating view of how CRABP1-positive malignant epithelial cells may be situated in the tumor microenvironment. CellChat-predicted APP-CD74 and PPIA-BSG networks with macrophage subpopulations are computational estimates based on transcriptomic data; they do not demonstrate direct ligand-receptor binding, cell-cell communication, or functional dependence. These candidate axes should therefore be tested experimentally before being assigned a mechanistic role in CRABP1-related thyroid carcinoma biology.

This study has several limitations. The single-cell and TCGA analyses were based on public datasets with differences in sample processing, clinical annotation, and cohort composition. The functional experiments were performed in selected thyroid cancer cell models, and broader validation would improve generalizability. The methylation analyses identify a CRABP1-associated epigenetic pattern, but the contribution of individual CpG sites to CRABP1 transcriptional control remains unresolved. The xenograft experiment used transient CRABP1 depletion, and stable models would provide stronger evidence for sustained *in vivo* effects. Despite these limitations, the study defines a CRABP1-positive malignant epithelial state, links CRABP1 loss to invasion-associated phenotypes, and connects CRABP1 dysregulation with methylation-related features in thyroid carcinoma.

## Conclusion

In conclusion, this study identifies a CRABP1-positive malignant epithelial cell state in PTC and associates lower CRABP1 expression with progression-associated clinicopathological features and altered transcriptional programs. Complementary thyroid carcinoma cell-line and xenograft experiments support an invasion-associated suppressive role for CRABP1, while remaining non-PTC-specific. Distinct public methylation observations and decitabine experiments provide a methylation-associated pharmacological context but do not establish cg02276974 as a regulatory site or demonstrate CRABP1-specific epigenetic causality. Predicted CellChat networks remain hypothesis-generating observations.

## Data Availability

The datasets presented in this study can be found in online repositories. The names of the repository/repositories and accession number(s) can be found in the article/Supplementary Material.

## Supplementary material

SUPPLEMENTARY FIGURE S1 Subclustering and copy number variation (CNV) profiling of epithelial cells. **(A)** UMAP projection of all epithelial cells, with each subcluster (C0–C9) distinguished by distinct colors as determined by unsupervised clustering. **(B)** InferCNV heatmap illustrating genome-wide CNV patterns across individual cells. Red indicates copy number amplifications and blue indicates copy number deletions, both relative to the diploid baseline, using macrophages as the reference (normal) population. **(C)** UMAP visualization of all epithelial cells, annotated into three categories: normal epithelial cells, CRABP1^+^ cancer cells, and CRABP1^−^ cancer cells.

SUPPLEMENTARY FIGURE S2 Exploratory GSEA of the external bulk RNA-seq cohort. **(A, B)** KEGG focal adhesion and tight-junction plots. **(C, D)** GO oxidative phosphorylation and ATP-synthesis-coupled electron-transport plots. These panels are exploratory pathway context. The legacy plot files do not retain FDR q values; conclusions based on these plots are not treated as confirmatory and require archived ranked-gene inputs and GSEA result tables for full FDR reporting.

## Glossary

ANOVA: Analysis of variance
CNV: Copy number variation
CpG: Cytosine-phosphate-guanine
CRABP1: Cellular retinoic acid binding protein 1
DAPI: 4′,6-Diamidino-2-phenylindole
DEG: Differentially expressed gene
DMEM: Dulbecco’s modified Eagle medium
DMSO: Dimethyl sulfoxide
EMT: Epithelial-mesenchymal transition
ES: Enrichment score
FBS: Fetal bovine serum
GAPDH: Glyceraldehyde-3-phosphate dehydrogenase
GEO: Gene Expression Omnibus
GO: Gene Ontology
GSEA: Gene set enrichment analysis
H&E: Hematoxylin and eosin
HRP: Horseradish peroxidase
KEGG: Kyoto Encyclopedia of Genes and Genomes
LNM: Lymph-node metastasis
MEXPRESS: Methylation and expression database for TCGA visualization
MSigDB: Molecular Signatures Database
NES: Normalized enrichment score
NK cell: Natural killer cell
oeCRABP1: CRABP1 overexpression
PTC: Papillary thyroid carcinoma
PVDF: Polyvinylidene difluoride
RIPA: Radioimmunoprecipitation assay
RNA-seq: RNA sequencing
RRID: Research Resource Identifier
RT-qPCR: Reverse-transcription quantitative polymerase chain reaction
SD: Standard deviation
SDS-PAGE: Sodium dodecyl sulfate-polyacrylamide gel electrophoresis
siCon: Non-targeting control small interfering RNA
siCRABP1: CRABP1-targeting small interfering RNA
siRNA: Small interfering RNA
TCGA: The Cancer Genome Atlas
TCGA-THCA: The Cancer Genome Atlas Thyroid Carcinoma cohort
THCA: Thyroid carcinomha
TPM: Transcripts per million
TSS: Transcription start site
UALCAN: University of Alabama at Birmingham Cancer Data Analysis Portal
UCSC Xena: University of California, Santa Cruz Xena browser
UMAP: Uniform manifold approximation and projection
